# Correction: Design, synthesis, and anti-plasmodial profiling of oxalamide-linked 4-aminoquinoline-phthalimide hybrids

**DOI:** 10.1039/d6md90001a

**Published:** 2026-01-21

**Authors:** Nikita Gupta, Anu Rani, Kewal Kumar, Raghu Raj, Vipan Kumar

**Affiliations:** a Department of Chemistry, Guru Nanak Dev University Amritsar-143005 India vipan_org@yahoo.com; b Department of Chemistry, Maharaja Ranjit Singh Punjab Technical University Dabwali Road Bathinda India; c P.G. Department of Chemistry DAV College Amritsar India

## Abstract

Correction for ‘Design, synthesis, and anti-plasmodial profiling of oxalamide-linked 4-aminoquinoline-phthalimide hybrids’ by Nikita Gupta *et al.*, *RSC Med. Chem.*, 2025, **16**, 4920–4928, https://doi.org/10.1039/d5md00425j.

The authors regret the omission of one of the authors, Anu Rani, from the original manuscript. The corrected list of authors and affiliations for this article is as shown above.

The Royal Society of Chemistry apologises for these errors and any consequent inconvenience to authors and readers.

